# Heterologous expression and characterization of laccase 2 from *Coprinopsis cinerea* capable of decolourizing different recalcitrant dyes

**DOI:** 10.1080/13102818.2014.913402

**Published:** 2014-07-10

**Authors:** Yong-Sheng Tian, Hu Xu, Ri-He Peng, Quan-Hong Yao, Rong-Tan Wang

**Affiliations:** ^a^ Institute of Agro-Biotechnology Research, Shanghai Academy of Agricultural Sciences, Shanghai, P.R.China; ^b^ Shanghai Ruifeng Agricultural Science and Technology Co. Ltd, Shanghai, P.R.China

**Keywords:** laccase, basidiomycete, *Coprinopsis cinerea* Okayama-7 #130, methyl orange, crystal violet, malachite green

## Abstract

The gene (CcLcc2) encoding laccase from the basidiomycete *Coprinopsis cinerea* Okayama-7 #130 was synthesized by polymerase chain reaction-based two-step DNA synthesis, and heterologously expressed in *Pichia pastoris*. The recombinant protein was purified by ammonium sulphate precipitation and nickel nitrilotriacetic acid chromatography. The molecular mass of CcLcc2 was estimated to be 54 kDa by denaturing polyacrylamide gel electrophoresis. The optimum pH and temperature for laccase catalysis for the oxidation of 2,2ʹ-azino-bis(3-ethylbenzothiazoline-6-sulphonate) (ABTS) were 2.6 and 45 °C, respectively. The *Km* values of the enzyme towards the substrates ABTS, 2,6-dimethoxyphenol (2,6-DMP) and guaiacol were 0.93, 1.02 and 28.07 mmol·L^−1^, respectively. The decolourization of methyl orange, crystal violet and malachite green, commonly used in the textile industry, was assessed. The decolourization percentage of crystal violet and malachite green was 80% after 4 h of reaction, and that of methyl orange was 50% at 4 h. These results show that the CcLcc2 has enormous potential for the decolourization of highly stable triphenylmethane dyes.

## Introduction

Wastewaters with substantial amounts of dyes from textile, paper or leather industries are often strongly coloured and detrimental to natural environment, even at very low dye concentrations.[1] These wastes reduce dissolved oxygen and light penetration in water and, therefore, affect the survival of aquatic life. Approximately 10% of the total dyes used in various textile processes worldwide are directly discharged without any treatment into the environment.[2,3] Therefore, pretreatment of these industrial effluents before release is of critical importance. Traditional physical–chemical processes, such as irradiation, precipitation and membrane-filtration, are usually expensive, inefficient and difficult to use.[4] Interest is now focused on better alternatives such as microbial biodegradation and biodecolourization of dyes. Several fungi and bacteria can degrade and decolourize dyes, especially some basidiomycetes that produce laccase, which is responsible for the degradation of these organic compounds,[5,6] have been isolated and characterized.[7–10]

Laccases or benzenediol:oxygen oxidoreductases are polyphenol oxidases that catalyse the oxidation of phenolic substrates and aromatic compounds to form various small molecular products.[11] Laccases show their unique function alone or in concert with other enzymes.[12] High-level laccase expression must be taken into account before it can be used commercially. It is well known that laccase expression level in some isolates is too low for industrial applications.[13] Heterologous expression in *Pichia pastoris* can meet these requirements, for it enhances the expression levels 10-, 100-, or even 1000-fold compared to the normal ones.[13,14]

*Coprinopsis cinerea* Okayama-7 #130 is an important ink cap basidiomycete that contains a large family of laccases (17 members). Preliminary studies indicate that 8 out of 17 members of this family exhibit enzymatic activity on 2,2ʹ-azino-bis(3-ethylbenzothiazoline-6-sulphonate) (ABTS) and 2,6-dimethoxyphenol (2,6-DMP).[15] However, decolourization of triphenylmethane and azo dyes by any member of this family, with the exception of a single or more species of bacteria, has not been reported.[4,16–20] In the present work, the gene of laccase, CcLcc2, was chemically synthesized and expressed in *P. pastoris*. After purification, its fundamental enzymological properties and application in decolourization of dyes were investigated.

## Materials and methods

### Organism, chemicals and dyes

Methyl orange (4-[4-(dimethylamino)phenylazo]benzene sulphonic acid sodium salt), crystal violet (hexamethylpararosaniline chloride), malachite green (N,N,N′,N′-tetramethy-l-4,4′-diaminotriphenylcarbenium chloride), ABTS, 2,6-DMP and guaiacol were obtained from Sigma-Aldrich commercially. The *P. pastoris* strain GS115 (his4) and plasmid pPIC9K were purchased from Invitrogen (San Diego, CA, USA). Unless otherwise stated, all other chemicals were from commercial sources and were of analytical grade.

### Chemical synthesis of CcLcc2, vector construction and transformation

The CcLcc2 gene without the N-terminus signal peptide and an added mating factor α signal sequence was chemically synthesized via the polymerase chain reaction (PCR)-based two-step DNA synthesis (PTDS) method.[21,22] Oligonucleotides of 60 bp lengths were synthesized by the Shanghai Sangon Biological Engineering Technology and Service Co. Ltd, China. The DNA fragment was cloned and sequenced after PCR amplification. The expression vector pYM7898 was constructed in our laboratory after inserting CcLcc2 into a modified pPIC9K (Invitrogen), in which the *Xho* I site in the G418 resistance cassette and the *Sac* I site in the AOX1 promoter were removed by site-directed mutagenesis. The 6*His-tag was added after the Kex2 protease cleavage site of the signal sequence. The plasmid pYM7898 (2 μg) was linearized by digestion with enzyme (*Sal* I) and then transformed into competent *P. pastoris* GS115 by electroporation (Bio-Rad Genepulser, Hercules, CA, USA). The transformants were screened on selective plates (1.34% yeast nitrogen base (YNB) without amino acids, 0.8 mol·L^−1^ sorbitol, 5% glucose and 2% agar). The colonies that appeared were subsequently screened by performing direct PCR to confirm integration of CcLcc2 into the *P. pastoris* GS115 genome.

### Expression and purification of recombinant CcLcc2 protein

A single *P. pastoris*-CcLcc2 colony was incubated into 50 mL of buffered glycerol-complex medium (1% yeast extract, 2% peptone, 1.34% YNB, 0.000004% biotin and 1% glycerol) at 28 °C with constant shaking (220 r·min^−1^), until the culture reached an OD_600_ of 4.0. The cells were then harvested (5000 r·min^−1^, 3 min), rinsed twice with sterilized water, and then resuspended to an OD_600_ of 1.0 in 200 mL of buffered minimal methanol (BMM) medium (100 mmol·L^−1^ potassium phosphate, pH 6.0, 1.34% YNB, 0.000004% biotin and 0.5% methanol). To induce the laccase gene expression, methanol was added every 24 h to a final concentration of 0.5%. Recombinant proteins in the culture supernatant were precipitated by 80% ammonium sulphate fractionation. The precipitate was resuspended with 10 mL of BMM solution and dialysed against the same buffer through sephadex G-15. The enzyme solution was applied to a HisTrap HP kit (Amersham Biosciences K.K., Tokyo, Japan) according to the manufacturer's instructions.

### Protein analysis and deglycosylation analysis

The laccase obtained after purification was analysed by 12% (w/v) sodium dodecyl sulphate polyacrylamide gel electrophoresis (SDS-PAGE), using the mini-protein gel electrophoresis system (Bio-Rad Lab, Hercules, CA, USA). The separated protein bands were stained with 0.2% Coomassie brilliant blue R-250. Protein concentrations were determined by the Bradford method, using bovine serum albumin as standard.[23] Deglycosylation of the natural laccase was performed using an N-glycosidase F deglycosylation kit (Roche, Penzberg, Germany), according to the manufacturer's instructions.

### Conditions of the laccase activity assay

Enzyme activity was determined by monitoring the oxidation of ABTS at 420 nm (ϵ_mM_ = 36.0 L·mmol^−1^·cm^−1^). The reaction mixture for the standard assay contained 1.6 mmol·L^−1^ ABTS in 190 μL of McIIvaine buffer (100 mmol·L^−1^ citric acid–Na_2_HPO_4_, pH 2.6) and 10 μL of purified enzyme. Assay mixtures were incubated at 30 °C for 10 min after initiating the reaction by the addition of enzyme. The reaction was terminated by the addition of 50 μL of 1.0 mol·L^−1^ NaF. One unit of enzyme activity is defined as the amount of enzyme that catalysed the oxidation of 1 μmol of ABTS per minute under the assay conditions.

### Effect of different parameters on enzyme activity

Optimal temperature and pH values for enzyme activity, temperature, pH stability, substrate (ABTS, 2,6-DMP and guaiacol) specificity and the effect of metal ions and other chemical reagents on laccase activity, were determined as described previously [5] with minor modifications.

The optimum temperature was investigated between 20 and 85 °C at 5 °C increments. Thermostability assays were determined by incubating the enzyme in McIIvaine buffer (pH 2.6) at 30, 40, 50 or 60 °C. Aliquots were removed at various times and assessed with ABTS as the substrate, as described above. The effect of pH on laccase activity was assayed using McIIvaine buffer at different pH ranges. The pH stability was examined by monitoring enzyme activity on ABTS in McIIvaine buffer after incubating the recombinant laccase in different pH buffers at 4 °C for 24 h. The effect of metal ions was also assessed. The enzyme was treated with 50 mmol·L^−1^ ethylenediaminetetraacetic acid (EDTA) for 30 min at 4 °C, applied to a sephadex G 15 column and eluted with BMM medium. The EDTA-treated enzyme was assayed in McIIvaine buffer (pH 2.6) containing 1.6 mmol·L^−1^ ABTS with or without 1 mmol·L^−1^ metal ions (e.g., CrCl_3_, MnSO_4_, FeSO_4_, Pb(NO_3_)_2_, MgSO_4_, CuSO_4_, ZnSO_4_, Cd(NO_3_)_2_, FeCl_3_, CoCl_3_ and AlCl_3_). Inhibitor studies were carried out in McIIvaine buffer containing different inhibitors, such as 0.1 and 1 mmol·L^−1^ dithiothreitol (DTT), 0.1 and 1 mmol·L^−1^ L-cysteine, 0.1 and 1 mmol·L^−1^ *p*-cumaric acid or 0.1 and 1 mmol·L^−1^ NaN_3_, 1% and 10% SDS or 1 and 10 mmol·L^−1^ EDTA. The substrate preference for ABTS (ϵ_420nm_ = 36.0 L·mmol^−1^·cm^−1^), 2,6-DMP (ϵ_468nm_ = 49.6 L·mmol^−1^·cm^−1^) and guaiacol (ϵ_465nm_ = 12.0 L·mmol^−1^·cm^−1^) was determined from Lineweaver–Burk plots of data obtained by measuring the reaction rates (three replicates) in McIIvaine buffer at 30 °C for 10 min. The substrate concentration ranges used were 0.07–3.84, 0.09–1.14 and 5.0–35.0 mmol·L^−1^, respectively.

### Effect of different parameters on dye decolourization

The decolourization of methyl orange, crystal violet and malachite green was assessed using the purified enzyme. The reaction mixture for the standard assay contained 10 μmol·L^−1^ dye, McIlvaine buffer and 50 μL of purified enzyme with or without a laccase mediator. Reaction was initiated by adding enzyme to the assay mixtures which was incubated at 30 °C for 10 min before, and then subsequently incubated in the dark. The time course of decolourization was determined at 20 min intervals in the first 2 h, and at 1 h intervals in the next 2 h by measuring the abosorbance at 470 nm for methyl orange, 590 nm for crystal violet and 620 nm for malachite green. Decolourization is defined as: Decolourization (%) = 100 × (Absorbance *t*_0_ − Absorbance *t_f_*) / Absorbance *t*_0_, where Absorbance *t*_0_ is the absorbance of the assay mixture before incubation; Absorbance *t_f_* is the absorbance after incubation. The amount of dye was calculated from standard curves of absorbance versus dye concentration. Control solutions contained heat-inactivated enzyme and blanks contained all of the components except the dye, which was substituted with the same volume of McIIvaine buffer. All the assays were carried out in triplicate.

The effect of pH on dye decolourization was studied by incubating the purified enzyme mixture in McIIvaine buffer (with 0.5 μmol·L^−1^ ABTS, 30 °C) adjusted to pH 2, 3, 4, 5, 6, 7 and 8. The effect of temperature was assayed by incubating the mixture at different temperatures from 20 to 80 °C at 10 °C increments at the optimum pH for each dye. To study the effect of dye concentration, 2.5, 5, 10, 20, 30 or 40 μmol·L^−1^ of final dye concentration was added in the reaction mixture, along with 0.5 μmol·L^−1^ ABTS at the optimum temperature and pH for each dye. The effect of ABTS as laccase mediator on dye decolourization was analysed using 0, 0.5, 1, 2.5, 5 or 10 μmol·L^−1^ ABTS in the reaction mixture at the optimum temperature and pH for each dye. To determine the effect of metal ions and laccase inhibitors on the decolourization, metal ions and inhibitors (at the same concentrations described above) were added to the reaction mixture. The mixture was incubated at the optimum temperature and pH for each dye, along with 0.5 μmol·L^−1^ ABTS as mediator.

### Sequence and data analysis

The Open Reading Frame (ORF) sequence of CcLcc2 was obtained from National Center for Biotechnology Information (NCBI) GenBank (Accession number BK004112) and analysed using the DNAMAN bioinformatics tool (version 6) (Lynnon Corporation, Quebec, Canada). The coding protein sequence of novel synthesized DNA sequence is the same as the protein sequence obtained from GenBank. Codon-usage processing was carried out using the database at http://www.kazusa.or.jp/codon. All graphs were constructed by OriginPro 7.5 (OriginLab, Northampton, MA, USA). All enzymological assays and dye decolourization tests were carried out at least in triplicate. Enzymological data in the graphs represent the averages of three replicates, and the error bars indicate the ranges of the values. Analytical results on dye decolourization varied by <5%.

## Results and discussion

### Purification of CcLcc2

Most white-rot fungi are capable of degrading or oxidizing a range of aromatic organic compounds with the aid of some enzymes, such as lignin peroxidase, manganese peroxidase, versatile peroxidase and laccase.[24] However, white-rot basidiomycetes possessing the same function only secrete one enzyme (laccase).[25] This indicates that laccase from basidiomycetes alone can decompose organic compounds. In addition, these laccases are available in edible mushrooms. These enzymes may be used in environmental bioremediation, as they are safe for humans, and remaining waste from mushroom cultivation after harvesting can be used as a source of the enzyme. Due to these potential applications, laccases have been studied most thoroughly in basidiomycetes.[26,27] In this work, laccase from C. *cinerea* was synthesized chemically and heterologously expressed in *P. pastoris* in active form. Laccases are not easily produced in large amounts as recombinant proteins.[13] In this work, the use of the α-factor signal peptide induced the extracellular secretion of recombinant proteins and facilitated subsequent recovery.

**Figure 1.**
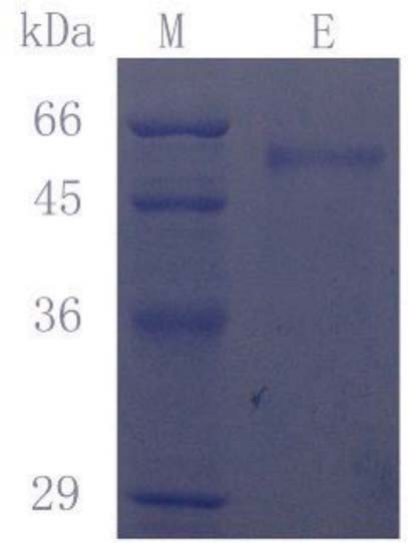
SDS-PAGE results of purified laccase (CcLcc2) secreted by *P. pastoris*. Lane M: protein molecular marker; Lane E: purified laccase.

After three days of culture, the supernatant exhibited the maximum laccase activity (data not shown). The purification steps for laccase are summarized in Table 1. The laccase was purified by (NH_4_)_2_SO_4_ fractionation from 10% to 80% and nickel nitrilotriacetic acid (Ni-NTA) affinity chromatography. The procedure resulted in ∼4.5-fold purification with 8.4% yield. The final specific activity of laccase was ∼27 U·mg^−1^. The purified enzyme showed a single protein band in SDS-PAGE (Figure 1). This indicates that no other byproduct proteins and laccase were extracellularly expressed by *P. pastoris* (lane E, Figure 1).

**Table 1.** Purification of secreted laccase from the 72 h culture supernatant.

Step*	Volume (mL)	Protein (mg·mL^−1^)	Activity (U·mL^−1^)	Sp. act. (U·mg^−1^)	Yield (%)	Purification (fold)
Crude supernatant	1000	0.42	2.56	6.10	100	1
(NH_4_)_2_SO_4_ fractionation	12	1.26	30.43	24.15	14.3	3.96
Ni-NTA affinity chromatography	5	1.59	43.22	27.18	8.4	4.46

*Data are values obtained in triplicate assays with variations of <5%.

### Physical characterization of CcLcc2

The molecular mass of CcLcc2 was around 54 kDa, as determined by SDS-PAGE assay. When the enzyme was treated with endoglycosidase H, there was no change in band size (result not shown). CcLcc2 has one potential N-glycosylation site in the sequence (Asn438), which is predicted by the NetNGlyc 1.0 server. This prediction proves that the extent of glycosylation of CcLcc2 is low.

### Laccase activity assay

#### Effects of pH and temperature on enzyme activity and stability

**Figure 2.**
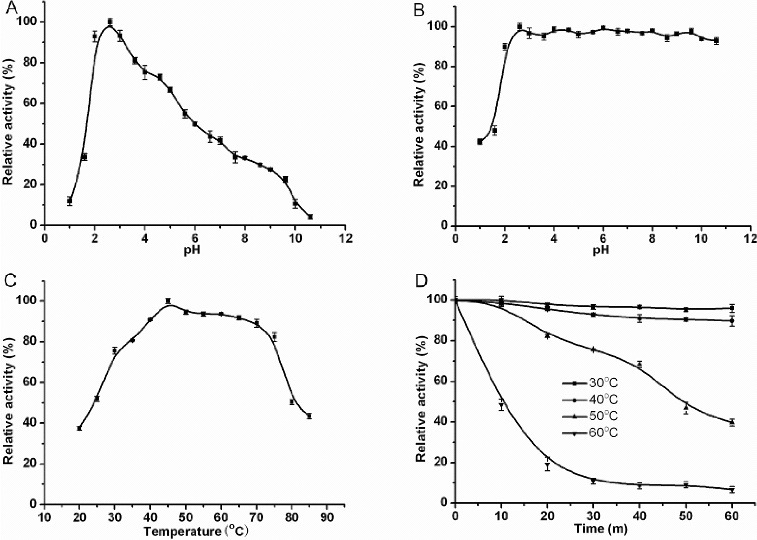
Properties of purified laccase: pH stability (A); pH dependence (B); thermal dependence (C); thermal stability (D). Reactions were stopped after 10 min, except in the thermal stability tests (D), with 50 μL of 0.25% NaF. Values are means ± SD of triplicate measurements.

The pH profile for laccase activity against ABTS showed a peak at pH 2.6, corresponding to the maximum activity (Figure 2(A)). When the effect of pH on enzyme stability was determined at 30 °C after different pH treatments at 4 °C overnight, the enzyme was found to be stable at pH 2.6–10.6 (Figure 2(B)). The optimum temperature of CcLcc2 was 45 °C (Figure 2(C)), and 90% activity was maintained at 40–70 °C. The thermal stability of laccase was determined by incubating the enzyme at pH 2.6 for 1 h and detecting its activity every 10 min. Laccase activity was hardly lost after incubating at 40 °C, except when the treatment temperature was increased to 60 °C, which caused 90% loss of laccase activity (Figure 2(D)). These results indicate that the purified enzyme has a wide optimum temperature range and good pH stability, which is usually required for industrial applications and is favourable for the development of biotechnological tools.

#### Substrate specificity

The conventional substrates oxidized by laccase, ABTS, 2,6-dimethoxyphenol and guaiacol, were used in the experiments designed to determine the enzymatic properties of CcLcc2. The kinetic parameters (Table 2) showed that CcLcc2 exhibited high activity with ABTS. The apparent *Km* value of this enzyme for ABTS was estimated to be 0.93 mmol·L^−1^. The apparent *Km* values for 2,6-DMP and guaiacol were 1.02 and 28.07 mmol·L^−1^, respectively. The relatively high *K*cat value for ABTS (846.78 s^−1^) suggests that ABTS is a preferred substrate for this enzyme.

**Table 2.** Substrate specificity and enzyme kinetics of the purified CcLcc2.

Substrate	*Km* (mmol·L^−1^)	*V*max (μmol·mg^−1^·min^−1^)	*K*cat (s^−1^)	*k*cat/*Km* (L·mol^−1^·s^−1^)
ABTS	0.93*	666.67	846.78	9.11 × 10^5^
2,6-DMP	1.02	57.47	73.00	7.16 × 10^4^
Guaiacol	28.07	2.46	3.12	1.11 × 10^2^

*Values obtained in triplicate assays with variations of <5%.

#### Effects of metal ions and inhibitors

**Figure 3.**
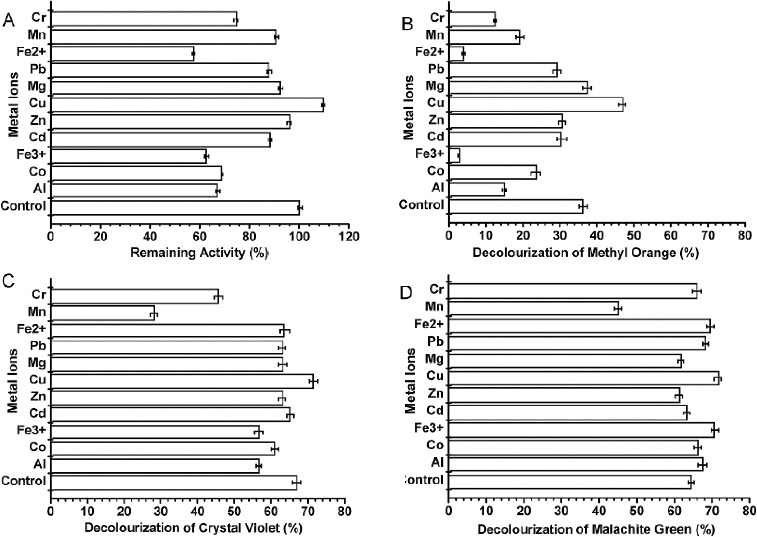
Effect of metal ions on purified enzyme activity (A) and decolourization of methyl orange (B), crystal violet (C) and malachite green (D). Metal ion concentrations were 1 mmol·L^−1^. Measurements were performed under optimum conditions. Reactions were stopped with 50 μL of 0.25% NaF after various durations. Values are means ± SD of three independent experiments.

The effects of metal ions and inhibitors on laccase activity were tested using ABTS as the substrate. The enzyme was inhibited by 1 mmol·L^−1^ Fe^2+^ (43% inhibition), Fe^3+^ (40% inhibition) and Co^3+^ (36%). In contrast, 1 and 5 mmol·L^−1^ Cu^2+^-activated laccase activity by 8% (Figure 3(A)) and 57% (data not shown), respectively.

**Figure 4.**
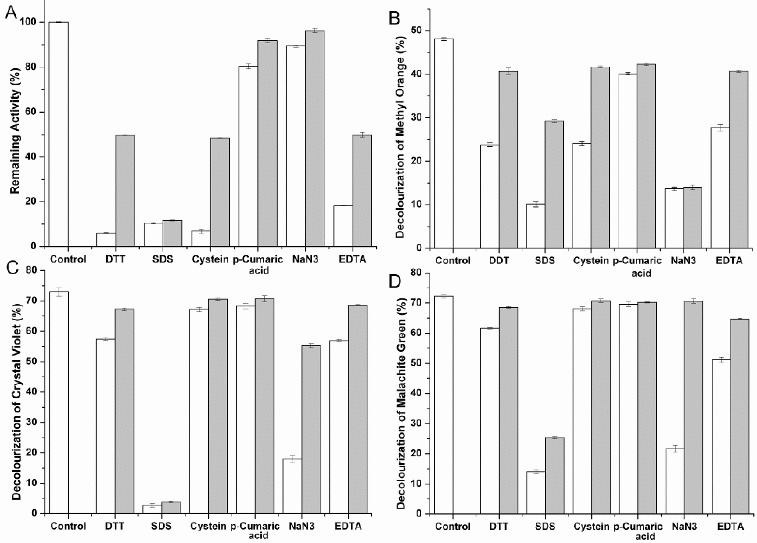
Effect of inhibitors on purified enzyme activity (A) and decolourization of methyl orange (B), crystal violet (C) and malachite green (D). White bars represent the effect of inhibitors at the higher of the two concentrations tested; grey bars represent the effect at the lower concentration (see Section Materials and Methods). Measurements were performed under optimum conditions. Reactions were stopped with 50 μL of 0.1% NaF after various durations. Values are means ± SD of three independent experiments.

The sensitivity of laccase to several laccase inhibitors was also determined (Figure 4(A)). Laccase activity was strongly inhibited by 1 mmol·L^−1^ DTT (94% inhibition), 1 mmol·L^−1^ L-cysteine (95%) and 1% SDS (90%). The metal chelator, 1 mmol·L^−1^ *p*-cumaric acid, caused a slight inhibitory effect (20%), whereas EDTA produced strong inhibition (80%).

### Decolourization of dyes

The purified enzyme prepared from the third day supernatant was evaluated for enzymatic decolourization activity.

#### Effect of pH

**Figure 5.**
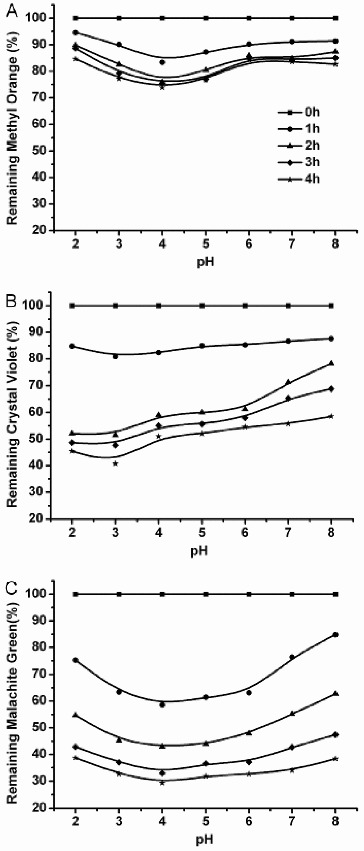
Effect of pH on decolourization of methyl orange (A), crystal violet (B) and malachite green (C) by the purified enzyme from *C. cinerea*. Reactions were stopped after 0, 1, 2, 3 or 4 h with 50 μL of 0.1% NaF. Data are values obtained in triplicate assays, with variations of <5%.

The optimum pH values for the decolourization of methyl orange, crystal violet and malachite green was 4.0, 3.0 and 4.0, respectively (Figure 5), which are slightly different from the optimum pH for oxidation of ABTS (Figure 2(A)). This result demonstrated that the pH optimum for laccase is dependent on the substrate. The purified enzyme mixture showed strong decolourization in the acidic pH range and weak decolourization at netural and alkaline pH. This result agrees well with the generally higher activity of laccase at low pH.[1]

#### Effect of temperature

**Figure 6.**
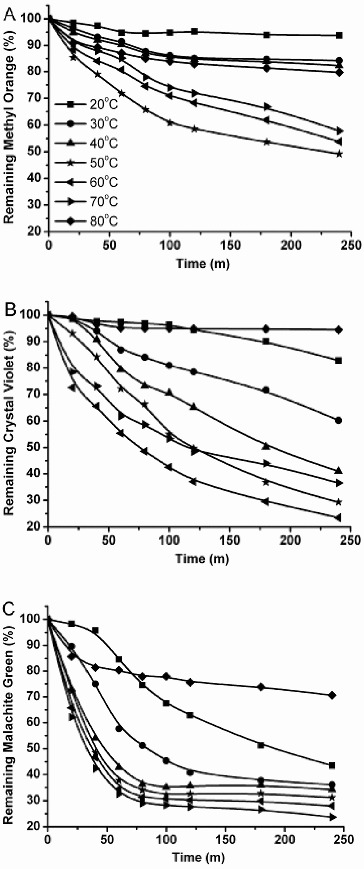
Effect of temperature on decolourization of methyl orange (A), crystal violet (B) and malachite green (C) by purified enzyme from *C. cinerea*. Reactions were stopped with 50 μL of 0.1% NaF after specific durations. Data are values obtained in triplicate assays with variations of <5%.

The influence of temperature on dye decolourization was investigated by incubating the reaction mixture at various temperatures. The optimum temperature of decolourization ranged from 50 to 70 °C. In the case of methyl orange, 15% dye decolourization was achieved at 2 h of incubation. When the temperature was >50 °C, the degree of decolourziation decreased to 50% of the maximum value (Figure 6(A)). With crystal violet, maximum decolourization of 80% was observed at 60 °C and it decreased to 40% at 30 °C (Figure 6(B)). Malachite green showed properties similar to those of crystal violet: the decolourization increased to 80% at 70 °C (Figure 6(C)).

#### Effect of dye concentration

The effect of dye concentration on the decolourization efficiency was determined with different initial dye concentrations (2.5–40 μmol·L^−1^) with a constant amount of purified enzyme. The decolourization decreased with the increase in dye concentration from 2.5to 40 μmol·L^−1^ for all dyes (Table 3). The behaviour observed here is similar to that of laccase oxidation of substrates, in which the rate of substrate oxidation increased with the substrate concentration until saturation. The maximum decolourization of methyl orange, crystal violet and malachite green at 4 h was 47.6%, 78.6% and 84.1%, respectively. The most effective decolourization was observed using crystal violet and malachite green as the substrate. The decolourization of methyl orange occurred more slowly than that of the other dyes during the initial period of the reaction, which indicates that CcLcc2 had more difficulty catalyzing the oxidation of azo dyes than of triphenylmethane dyes. The latter may need relatively strong laccase activity or a longer reaction time.

**Table 3.** Decolourization of three dyes at different dye concentrations*.

	Decolourization (%)								
	Methyl orange			Crystal violet			Malachite green		
Dye concentration (μmol·L^−1^)	1 h	2 h	4 h	1 h	2 h	4 h	1 h	2 h	4 h
2.5	25.9	50.5	76.9	63.7	81.4	96.3	80.5	90.2	96.5
5	16.1	41.2	53.5	55.1	77.8	89.9	75.5	86.1	90.9
10	10.3	35.6	47.6	44.2	67.1	78.6	69.2	82.2	84.1
20	5.3	21.1	44.7	41.2	57.9	73.2	60.4	79.3	82.1
30	2.9	8.6	17.9	22.8	45.7	65.6	44.6	70.2	73.5
40	1.3	5.2	11.4	16.0	31.8	55.8	38.7	58.3	64.2

*Effects of dye concentration on decolourization by purified enzyme. The specific decolourization measurements were performed at optimum pH and temperature and concentration of redox mediators. Data are values obtained in triplicate assays with variations of <5%.

Although the two triphenylmethane dyes were oxidized in the presence of recombinant laccase to the same extent, the decolourization processes were different. Crystal violet was more resistant to decolourization than malachite green. At the start, the decolourization of crystal violet was slow and increased with time, whereas malachite green was decolourized rapidly and barely any decolourization took place in the next reaction (Figure 6(B) and 6(C)). This may be due to differences in the chemical structure of the dyes. Moreover, the decolourization activity towards crystal violet was inhibited when the dye concentration was 20 μmol·L^−1^, whereas 30 μmol·L^−1^ malachite green resulted in inhibition. This result further indicates that malachite green is more easily decolourized than crystal violet. A similar result with other bacterial species has been observed.[28] On the contrary, there are certain bacterial species that can decolourize crystal violet more easily than malachite green.[18]

#### Effect of redox mediator

**Figure 7.**
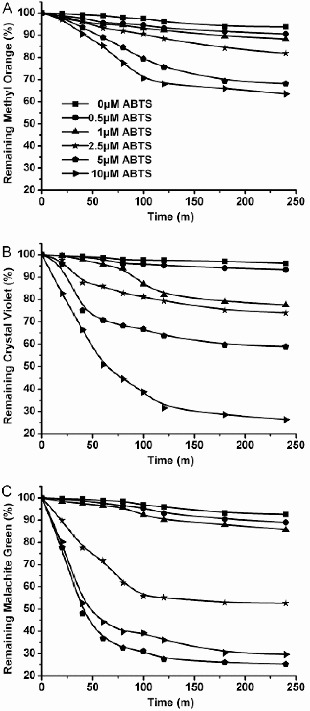
Decolourization of methyl orange (A), crystal violet (B) and malachite green (C) by purified enzyme from *C. cinerea* with redox mediators at various concentrations. Dyes were incubated with CcLcc2 and different ABTS concentrations at their optimum pH and temperature. Data are values obtained in triplicate assays with variations of <5%.

All tested dyes were poorly decolourized (∼10%) by purified enzyme alone. However, in the presence of a redox mediator (ABTS), the purified enzyme achieved efficient decolourization, >50% in 4 h (Figure 7). These results agree with previous studies on other mediators.[7,29] Small increases in the rate of decolourization of dye were observed (Figure 7). With the increase in ABTS concentration up to the dye concentration, decolourization rates did not decrease, except that of malachite green (Figure 7(C)). The inhibition of decolourization of malachite green could be explained by the high concentration of ABTS combining with malachite green. Many investigations have demonstrated that adding certain redox-active compounds to the reaction system can enhance the decolourization of dyes effectively and efficiently. The divalent cation ABTS^2+^ (the oxidation product of laccase) may have a role in the oxidation of the dyes as an intermediate oxidant. Thus, it could be suggested that the decolourization of the tested dyes was indirect and the process was caused by the oxidizing intermediates produced by the laccase enzyme.

#### Effect of metal ions and inhibitors

Industrial wastewaters are complex mixtures that may contain dyes, salts, metal ions, chelators, precursors and so on.[30] Some of these substances may deactivate laccase. Previous studies have demonstrated that there are limitations to the ability of laccase to decolourize effluents. Therefore, the effects of metal ions and laccase inhibitors on laccase decolourization were investigated in our work.

Metal ions and some potential laccase inhibitors (e.g., DTT, SDS, L-cysteine, *p*-cumaric acid, NaN_3_ and EDTA) were tested for their effect on dye decolourization. The effects of the metal ions are shown in Figure 5. Decolourization of methyl orange (Figure 3(B)) was severely impacted by Fe^2+^ and Fe^3+^. However, the same ions had little effect on the decolourization of crystal violet (Figure 3(C)) and malachite green (Figure 3(D)).

The effects of putative laccase inhibitors on dye decolourization are shown in Figure 4. Decolourization of methyl orange (Figure 4(B)) in the presence of NaN_3_ was strongly inhibited even at 1 mmol·L^−1^ NaN_3_. The effect of each inhibitor was generally proportional to its concentration. The effect of other inhibitors on the decolourization of crystal violet (Figure 4(C)) and malachite green (Figure 4(D)) in the presence of NaN_3_ was generally different from that on the decolourization of methyl orange. Other inhibitors (except SDS) slightly inhibited the decolourization of crystal violet and malachite green. SDS severely inhibited the decolourization of crystal violet and malachite green and had relatively little effect on methyl orange decolourization.

## Conclusions

In this work, we demonstrated that the expression of the laccase gene (CcLcc2) from the basidiomycete C. *cinerea* Okayama-7 in the methylotrophic yeast *P. pastoris* produces an extracellular laccase which is stable and active in the presence of moderate amounts of metal ions and organic solvents. This enzyme has a good decolourization capacity towards three textile dyes. This capacity can be enhanced by the addition of mediators such as ABTS. In view of the results obtained in the present work, it is clearly indicated that this laccase possesses important properties for industrial applications. Characterization of other functional members of this laccase family is underway and the catalytic mechanism will be subject to future studies.
